# Asymptomatic Tumor Thrombus in the Left Atrium from Squamous Cell Carcinoma

**DOI:** 10.1155/2021/4256471

**Published:** 2021-12-21

**Authors:** Nick Huang, Christina DiCorato, Basel Abuzuaiter, Adriana May, Swati P. Deshmane, Debanik Chaudhuri, Stephen Graziano, Harvir Singh Gambhir

**Affiliations:** ^1^Department of Medicine, SUNY Upstate Medical University, Syracuse, NY, USA; ^2^Department of Pathology, SUNY Upstate Medical University, Syracuse, NY, USA; ^3^Department of Radiology, SUNY Upstate Medical University, Syracuse, NY, USA; ^4^Department of Hematology and Oncology, SUNY Upstate Medical University, Syracuse, NY, USA

## Abstract

A 67-year-old female patient presented asymptomatically for further evaluation of a chest mass. Other than significant smoking history, the patient had been healthy with a recently treated case of uncomplicated pneumonia. The mass originated in the aortopulmonary window of the left mediastinum and invaded proximally into the left superior pulmonary vein and subsequently into the left atrium. The mass protrusion into the mitral valve occupied 50% of the left atrium space but showed no clinical symptoms of a valvular blockade. Poorly differentiated squamous cell carcinoma was identified upon biopsy. These findings of a primary lung tumor with atrial extension in an asymptomatic patient point to the importance of age-appropriate screening and standardization treatment modalities.

## 1. Introduction

The evaluation of cardiac masses frequently presents diagnostic challenges due to a combination of their broad differential, imaging challenges, and rarity, with a prevalence of 0.001 to 0.03% of primary tumors [1], and a 20–40 higher incidence of secondary tumors [2]. When the tumor is an extension of lung cancer, there is no standardized approach to its management. Still, typically surgical resection is more common if there exists an intra-atrial extension of tumor thrombus [3]. We present a case of a rare presentation of lung cancer in an asymptomatic healthy female patient found to have a tumor thrombus originating in the pulmonary veins and extending into the left atrium.

## 2. Case Report

**Figure 1:**
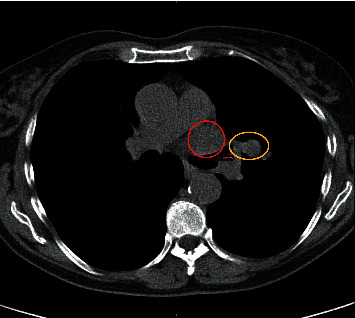
Screening chest CT scan that originally identified the 19 × 22 mm nodule in the left upper lobe of the lung (red circle). Left hilar adenopathy was also noted on presentation (yellow circle). Intralobular septal thickening was also noted.

The patient is a 67-year-old previously healthy female with a 55 pack-year smoking history presented with an indication of a mediastinal mass that was incidentally found on a chest radiograph when she developed pneumonia that resolved without complications several months prior. She denied any weight loss, fever, chills, or night sweats. After 30 years of not being seen by a healthcare provider, she subsequently established with a primary care physician. At that time, age-appropriate cancer screening was initiated, including a low-dose CT (computed tomography) scan of the thorax, which identified a 19 × 21 mm nodule, left hilar lymphadenopathy, and intralobular septal thickening in the left upper lobe (Figure 1) suspicious for pulmonary venous obstruction.

**Figure 2:**
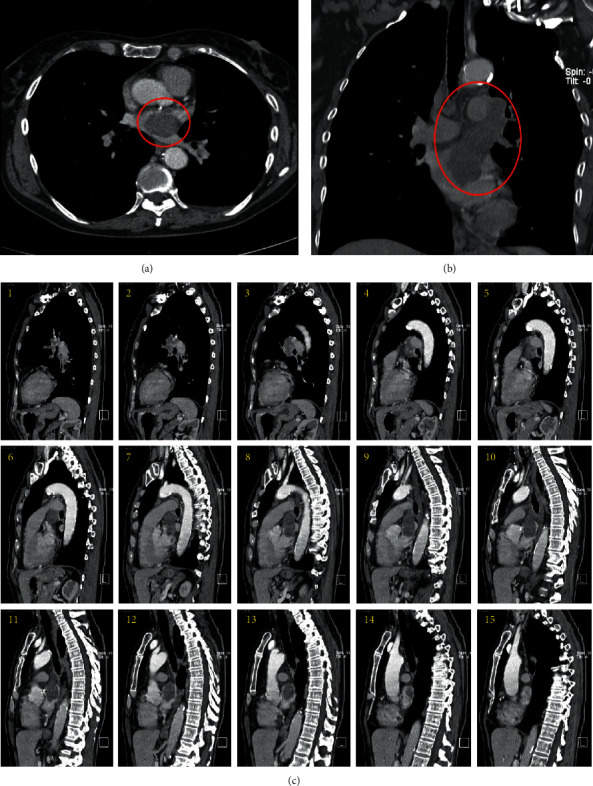
CT thorax with contrast sagittal (a) and coronal (b) representative views of the patient's mass measuring 3.3 × 2.4 cm. A sequence depicting the origination of the mass as it invades the left superior pulmonary vein before its extension in the left atrium (c).

**Figure 3:**
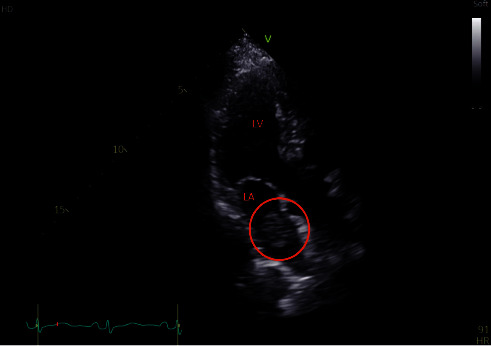
Transthoracic echocardiography (TTE) image of a mobile ecodensity measuring 3.0 cm × 1.6 cm. Doppler radar of the flow (not shown here) did not show obstruction of flow, which contributed to the patient's asymptomatic status.

On direct admission to the hospital for further workup of the pulmonary mass, the patient was hemodynamically stable during the admission with stable vitals and moderate hypertension of 150/90 mmHg. CT Thorax with contrast verified a mass in the aortopulmonary window measuring 3.3 × 2.4 cm (Figures 2(a) and 2(b)) and extending proximally into the left superior pulmonary veins and subsequently the left atrium (Figure 2(c)). Transthoracic echocardiography (TTE) revealed a left ventricular ejection fraction (LVEF) of 61% with a 3.0 cm × 1.6 cm mobile echo density with a poorly defined variegated surface visualized in the left atrium attached to the roof of the atrium (Figure 3). The hypoechoic mass moves slightly with the cardiac cycle but remains within the left atrium. The mass appears to be projecting in from the right pulmonary vein but is not clearly defined. In some echocardiographic views, the mass occupies more than 50% of the left atrium and has an undulating thrombus on the surface of the tumor.

**Figure 4:**
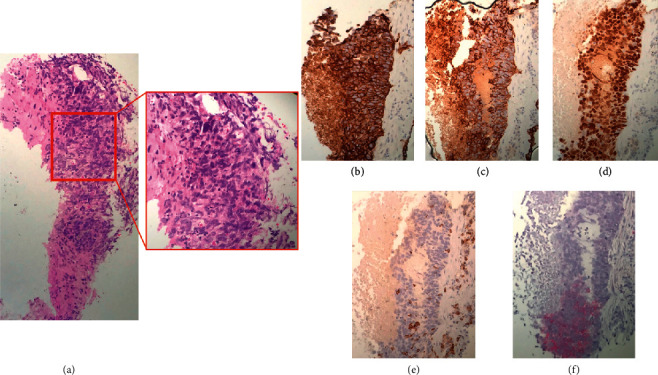
Histological sections of the left hilar mass. (a) Sections show cores of lung parenchyma replaced by a malignant neoplasm with significant pleomorphism, nuclear vacuoles, and abundant necrosis. Immunostaining showed positivity for (b) cytokeratin AE1/AE3, (c) CAM5.2, (d) p63, (e) CD5 (patchy), and (f) CD117 (patchy). The tumor cells are negative for PAX-8, napsin, TTF-1, CK7, CK20, S100, TTF-1, synaptophysin, chromogranin, and CDX-2 (not shown). This immunoprofile is supportive of a thymic origin and was diagnosed as poorly differentiated squamous cell carcinoma. There were not enough tissues to perform ancillary molecular studies.

Initial workup included tumor staging with CT abdomen and pelvis, magnetic resonance imaging (MRI) of the brain, and nuclear bone scan, all of which returned negative for metastatic disease. An adrenal mass was incidentally found on CT abdomen and follow-up MRI abdomen with contrast established; it was a benign adrenal adenoma. Clinically, the patient appeared healthy and did not have any symptoms or imaging findings suggestive of metastatic disease. The patient reported no weight loss, fevers, chills, changes in appetite, chest pain, or SOB. The patient's complete blood count (CBC) and basic metabolic profile (BMP) were unremarkable, except for mild hypokalemia of 3.2. A multidisciplinary approach was utilized: neurology for assessing anticoagulation and stroke prevention due to tumor thrombus involvement: thoracic/cardiac surgery for surgical evaluation; pulmonary for tumor evaluation and management; and interventional radiology for sample biopsy. Interventional radiology CT-guided percutaneous biopsy obtained a pathology sample of the left hilar mass that was identified as a poorly differentiated squamous cell carcinoma. The tumor cells had an immune profile that was supportive of thymic origins with positive staining for cytokeratin ae1/ae3, CAM5.2, p63, CD5 (patchy), CD117 (focal and patchy), and negative staining for PAX-8, napsin, TTF-1, CK7, CK20, S100, synaptophysin, chromogranin, and CDX-2 (Figure 4).

After discussing with multiple disciplines (radiation, medical oncology, and surgery teams), the plan was to initiate inpatient chemotherapy with concurrent radiation therapy. The patient was transferred to the oncology inpatient service for further management. Her final staging was T4N3M0, Stage 3C. Evaluation by the surgical specialties deemed the tumor to be nonresectable due to location and complexity. As such, following the data obtained from the Pacific trials [4], the patient was subsequently started on 6000 cGy radiation with concurrent turbotaxol and subsequently followed up with one year of durvalumab. On patient follow-up visits two months after initiating therapy, she is tolerating treatments with minimal adverse effects. Follow-up CT thorax demonstrates a decreased lung mass down to 16mm × 8mm (from 19 × 22 mm).

## 3. Discussion

The most common causes of intracardiac tumors are hepatocellular carcinoma, renal cell carcinoma (RCC), and adrenal cortical carcinoma (ACC) [5]. Urgent evaluation and complete staging are essential aspects of management with cardiac CT and magnetic resonance imaging (MRI) as the most commonly used methods [1]. In the case of our patient, the workup was all negative. Ultimately a biopsy was obtained, which determined the mass as a Stage IIIC squamous cell carcinoma of the lung with possible thymic origins. What makes this case unique is the asymptomatic presentation of a sizeable nonobstructive mass in an otherwise healthy female patient of relatively young age compared to previous case reports with typical age ranging from the mid-70s to mid-80s.

Lung cancer is the second most common cancer and is responsible for most cancer-related deaths in the US and worldwide [6]. Lung cancer invasion into the heart is a rare phenomenon, with only a few reported cases worldwide in the last decade [3, 7–10]. Most of these cases present with symptomatic patients to varying degrees, from the potential of embolization causing stroke [10], bowel infarction, and ischemic leg [11] to the left ventricular outlet obstruction resulting in sudden death [8, 9]. There is still no standardized approach to such masses given these devastating complications, although surgery before chemotherapy is generally advocated [3].

In our case, a healthy female patient in her mid-60s presented with an asymptomatic mediastinal mass and a mobile echo density in the left atrium. On TTE, the mass is fairly large with a poorly defined structure, unlike cardiac myxomas. Our initial differential was broad and included anatomical variations, implantable devices, thrombus, vegetations or other infectious etiologies, traumatic scar (patient had a car accident history), artifacts, and tumors. Among tumors, lung cancer has the highest incidence of metastasis to the heart, followed by breast cancer, malignant melanoma, leukemia, and malignant lymphoma [12, 13]. Additionally, on the differential were myxomas, fibromas, and rhabdomyomas. Due to the tumor's extension, we also suspected sarcoma as a malignant tumor source. Lastly, metastatic workup included CT, MRI, and NM bone scans returned negative; the diagnosis ultimately depended on the biopsy result. A biopsy of the left hilar mass was obtained with the help of interventional radiology, and subsequent staining was completed.

Due to the number of possible complications that can arise from the mass and its associated treatment, a multidisciplinary approach is necessary to identify and classify the tumor and its subsequent therapy steps. The tumor was classified as T4N3M0, Stage IIIC, due to the lack of distant metastasis. The thymic origins suggested possible lymphoma, but after reviewing the histological slides, the diagnosis of squamous cell carcinoma or NSCLC was made, and treatment was planned accordingly. Under the current NCCN guidelines, the patient was treated with chemoradiation per the Pacific trials and will be evaluated for surgical candidacy in the future. At the time of this manuscript submission, the patient is doing well and is tolerating her treatments. Tumor shrinkage was noted on chest CT after two months of therapy. This presentation helps to signify the importance of age-appropriate lung cancer screening with low-dose CT in those with significant smoking history, which recently has been shown to have evidence-based mortality reduction [14].

## 4. Conclusion

Lung cancer with tumor thrombus can be a rare and severe complication with involvement of the left atrium. Especially with debilitating complications like stroke and infarction, it becomes prudent for age-appropriate screening and early detection of these tumors. The prevalence of squamous cell carcinoma of the lung presenting as tumor thrombus in the left atrium is a rarity. Therefore, we need further studies to learn about more multidisciplinary team management strategies and treating such masses. Here, we report a case of an asymptomatic left atrial invasion from squamous cell carcinoma of the lung.

## Data Availability

All data are made available in the publication.
